# A BIM-Based Digital Twin Framework for Urban Roads: Integrating MMS and Municipal Geospatial Data for AI-Ready Urban Infrastructure Management

**DOI:** 10.3390/s26030947

**Published:** 2026-02-02

**Authors:** Vittorio Scolamiero, Piero Boccardo

**Affiliations:** 1 Department of Civil, Building and Environmental Engineering (DICEA), Sapienza Università di Roma, Via Eudossiana, 18, 00184 Rome, Italy; 2 Interuniversity Department of Regional and Urban Studies and Planning (DIST), Polytechnic of Torino, Viale Pier Andrea Mattioli, 39, 10125 Torino, Italy; piero.boccardo@polito.it

**Keywords:** digital twin, urban road network, road asset management, BIM, MMS, LiDAR, urban mapping

## Abstract

Digital twins (DTs) are increasingly adopted to enhance the monitoring, management, and planning of urban infrastructure. While DT development for buildings is well established, applications to urban road networks remain limited, particularly in integrating heterogeneous geospatial datasets into semantically rich, multi-scale representations. This study presents a methodology for developing a BIM-based DT of urban roads by integrating geospatial data from Mobile Mapping System (MMS) surveys with semantic information from municipal geodatabases. The approach follows a multi-modal (point clouds, imagery, vector data), multi-scale and multi-level framework, where ‘multi-level’ refers to modeling at different scopes—from a city-wide level, offering a generalized representation of the entire road network, to asset-level detail, capturing parametric BIM elements for individual road segments or specific components such as road sign and road marker, lamp posts and traffic light. MMS-derived LiDAR point clouds allow accurate 3D reconstruction of road surfaces, curbs, and ancillary infrastructure, while municipal geodatabases enrich the model with thematic layers including pavement condition, road classification, and street furniture. The resulting DT framework supports multi-scale visualization, asset management, and predictive maintenance. By combining geometric precision with semantic richness, the proposed methodology delivers an interoperable and scalable framework for sustainable urban road management, providing a foundation for AI-ready applications such as automated defect detection, traffic simulation, and predictive maintenance planning. The resulting DT achieved a geometric accuracy of ±3 cm and integrated more than 45 km of urban road network, enabling multi-scale analyses and AI-ready data fusion.

## 1. Introduction

Road networks are among the most critical infrastructures in cities, directly shaping mobility, safety, and the sustainability of urban life [1]. Their continuous deterioration due to traffic loads, weathering, and aging represents a significant financial and social burden, particularly in dense metropolitan contexts. Efficient monitoring and management of road assets therefore play a decisive role in supporting resilient and sustainable urban environments [2]. Yet, traditional inspection and maintenance strategies are still largely based on manual surveys or isolated sensing campaigns, often resulting in fragmented datasets, high costs, and delayed interventions. This critical role increasingly requires integrated digital representations capable of supporting both network-scale planning and asset-level decision-making.

In recent years, DT technology has emerged as a transformative paradigm for infrastructure management, allowing the creation of dynamic, data-driven replicas of physical assets [3]. In the road domain, DTs are increasingly applied to support condition monitoring, predictive maintenance, and lifecycle planning, often enhanced by artificial intelligence (AI) techniques [4]. By integrating heterogeneous data into semantically rich, continuously updatable models, DTs can provide decision-makers with a comprehensive view of infrastructure performance in real time [5]. Despite this potential, most existing road DT initiatives are limited to specific highway corridors or experimental testbeds. However, in practice, most existing DT implementations for road infrastructure remain fragmented, often focusing on isolated assets, experimental corridors, or highway-scale scenarios, with limited attention to dense urban road networks. Very few initiatives have addressed the complexity of dense urban networks, revealing a persistent gap in the deployment of city-scale road DTs, where issues of data integration, interoperability, and institutional governance remain challenging. Urban contexts introduce additional challenges related to heterogeneous data sources, interoperability between GIS and BIM environments, and institutional data governance, which are still insufficiently addressed in current DT research.

Digitalization is essential in urban contexts for improving efficiency, enhancing safety, and enabling smarter traffic management. Digitizing road infrastructure represents a transformative step toward modernizing transportation networks and promoting urban sustainability [6]. Urban roads, as the backbone of mobility, are considered critical infrastructure because their disruption can significantly affect emergency services, commerce, public transport, and daily life. Failures or degradation in road networks can cascade into economic losses, safety hazards, and reduced resilience of the urban system. By integrating geospatial data, IoT sensors, and artificial intelligence, cities can achieve real-time monitoring, predictive maintenance, and data-driven decision-making, enhancing the robustness of these critical networks. Within this broader digitalization process, DT represent the operational layer that connects raw sensing data with simulation, analytics, and decision-support capabilities. DTs of urban roads provide a powerful tool in this context: by creating a virtual replica of the physical network, they enable continuous condition monitoring, simulation of traffic scenarios, and proactive maintenance planning, ensuring resilience and reliability [5]. Aligning digitalization efforts with global sustainability goals is particularly significant: optimized resource usage and reduced environmental impact contribute to better air quality and lower carbon emissions. For instance, energy-efficient highway lighting and traffic flow optimization not only decrease operational costs but also support eco-friendly urban development, while ensuring that the urban road network remains safe, reliable, and resilient in the face of growing traffic demand and potential disruptions.

Addressing the aforementioned gap, the present study proposes a BIM-based DT for urban roads that integrates MMS point clouds with authoritative municipal geospatial datasets. This integration enhances both geometric accuracy and semantic richness, creating AI-ready information models that are directly applicable to urban infrastructure management. By fusing detailed survey data with existing municipal sources, the proposed workflow ensures that road models are not only precise in their geometry but also enriched with administrative and functional attributes, thus supporting advanced analytical and decision-making processes.

The contributions of this work are threefold:

- It develops a workflow for transforming MMS and municipal geospatial data into a unified BIM-based DT of urban roads.
- It demonstrates how this integration enhances the information content and AI-readiness of road models, facilitating condition assessment and management tasks.
- It highlights the potential of scaling DT approaches to the city level, addressing a critical gap in current road DT research.

Positioned within the broader vision of city-scale DTs, this study proposes an interoperable and multi-scale framework specifically designed for urban road networks, enabling the integration of geometric, semantic, and administrative data into a unified environment. Importantly, the present work does not implement a fully dynamic DT with real-time sensor ingestion or bidirectional communication. Instead, it provides a DT-ready information environment, where heterogeneous datasets (MMS, GIS, and municipal geodatabases) are harmonized within a semantically rich BIM–GIS structure. This static but interoperable foundation serves as the baseline upon which real-time data streams and API-based functionalities may be integrated in future developments.

While multi-modal (LiDAR, imagery, GIS), multi-scale (LoD 200–400), and multi-level (city–road–asset) integration are essential characteristics of many DT architectures, it is important to clarify that these components alone do not constitute a fully functional DT. Rather, they establish the foundational data infrastructure necessary for enabling continuous updates, real-time communication, and decision-support processes.

Future evolutions of this framework could incorporate IoT sensor networks, enabling near-real-time monitoring of traffic, pavement conditions, and environmental variables. Such developments would allow the DT to evolve into a dynamic, operational decision-support tool; however, challenges related to data synchronization, sensor calibration, and network infrastructure must be addressed.

Finally, while the present methodology enhances geometric and semantic integration, it still relies on periodic survey data and partial manual intervention. Scaling this approach to entire urban networks will require careful considerations of computational resources, data governance strategies, and privacy regulations.

### 1.1. Aim of This Paper

This research addresses the growing need for the digitalization of the urban environment, focusing specifically on one of the most critical infrastructures within the city: the road network system. The main objective is to develop a methodological framework for the creation of a digital representation of the road infrastructure, integrating BIM with data derived from authoritative geodatabases, GIS platforms, and urban mapping activities. This integration aims to enhance the interoperability between geometric, semantic, and spatial information, enabling a more comprehensive and data-driven management of the urban road network.

In this context, the integration can be described as a multi-source, multi-scale data fusion process that connects geometric modeling (BIM), spatial referencing (GIS), real-world data acquisition (Urban Mapping), and institutional data reliability (Authoritative Geodatabase) within a single interoperable environment. Together, these elements form the backbone of a DT or digital representation of the urban road system—where BIM provides structure and semantics, GIS ensures spatial consistency, urban mapping supplies geometric reality, and the authoritative geodatabase guarantees institutional reliability.

BIM is employed to model the physical and semantic characteristics of the road infrastructure. It translates the geometric information obtained from the mapping phase into parametric objects enriched with attributes describing materials, condition indicators, and functional components. The BIM environment thus supports the creation of an information-rich digital model that can be continuously updated. GIS establishes the spatial framework in which the road infrastructure is located. It allows the georeferencing, visualization, and analysis of all spatial layers, supporting the integration of multiple data sources at different scales. Within this environment, the road network is spatially connected to other urban elements such as utilities, administrative boundaries, and land-use information. Urban Mapping provides the geometric foundation of the digital model. It includes the acquisition and processing of high-resolution spatial data, such as LiDAR point clouds and photogrammetric imagery, derived from MMS. These data deliver an as-built representation of the road surface and surrounding urban features. The Authoritative Geodatabase complements these components by providing validated and standardized data from institutional sources. It ensures that the resulting model adheres to official classifications, nomenclatures, and spatial reference systems, enhancing data reliability and interoperability.

The integration of these four elements—geometric (Urban Mapping), spatial (GIS), semantic (BIM), and administrative (Authoritative Geodatabase)—forms the foundation for a consistent and multi-source digital twin of the urban road network. This integrated environment enables both spatial analysis and information modeling, supporting the monitoring, management, and maintenance of urban infrastructure.

Although MMS- and LiDAR-derived point clouds enable detailed reconstruction, many workflows still rely on human supervision for classification and parameterization. Future work could leverage AI-based approaches, including deep learning models for automated road defect detection and semantic enrichment. Challenges such as the creation of robust training datasets, generalization to diverse urban contexts, and computational costs need to be addressed to scale these methods for city-wide deployment.

### 1.2. State of the Art of DTs Application on Road Infrastructure

**Figure 1:**
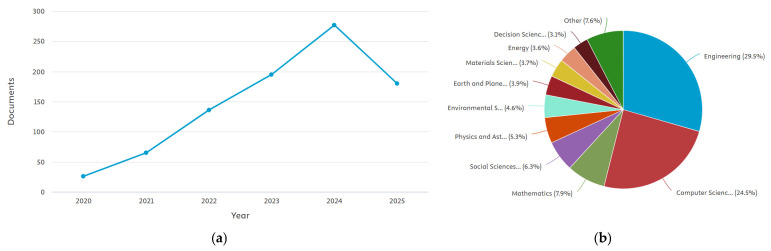
Number of publications per year retrieved from the Scopus database using the keyword “digital twin road.” (**a**) The chart illustrates the growth of research activity in road digital twins over time, focusing on the last five years. (**b**) The chart illustrates the documents by the subject area.

DT technology has emerged over the past decade as a transformative paradigm in infrastructure management, enabling the creation of dynamic, data-driven replicas of physical assets that can be continuously updated with sensor streams and historical records. Within the road domain, interest in DTs is steadily increasing, as illustrated in Figure 1, driven by the need for improved condition monitoring, predictive maintenance, and more sustainable management of transport networks. Digitizing road infrastructure also represents a critical step toward urban resilience, as road networks are essential for emergency services, public transport, commerce, and daily mobility. Disruptions or failures in these networks can have cascading effects on safety, economic activity, and overall urban functionality, highlighting the need for robust, data-driven management solutions.

As shown in Figure 1, research interest in the application of DT technology to roads has grown rapidly in recent years. Publications retrieved from the Scopus database increased markedly after 2020, reflecting the broader diffusion of DT concepts across the infrastructure and civil engineering domains. Between 2020 and 2024, the number of documents using the keyword “digital twin road” rose from 26 to 277 documents, confirming the accelerating pace of research and development in this field. However, a closer inspection of the subject areas reveals that most studies focus on transportation engineering, computer science, and construction management, while relatively few address the integration of DTs within the urban context. This imbalance highlights that, despite the growing maturity of DT applications for highways and controlled testbeds, city-scale road systems remain a largely unexplored domain.

Recent studies demonstrate a significant evolution in DT research, transitioning from theoretical frameworks to practical applications in condition monitoring, predictive maintenance, and transport planning, with a growing emphasis on AI-driven analytics. DTs have been successfully applied to road pavement monitoring and predictive maintenance [7], supported by enabling technologies for road engineering and lifecycle applications [8]. Systematic reviews highlight their role in improving the resilience and sustainability of rail and road networks [9], while advanced communication technologies such as 6G facilitate real-time data acquisition and connectivity essential for DT-enabled infrastructures [10]. Current surveys emphasize DT applications across transportation infrastructure, addressing challenges and future directions for AI-driven analytics [11], and demonstrate the convergence between physical and digital systems that underpins predictive and adaptive urban transport solutions [12]. In addition, recent work illustrates how AI, IoT, and BIM integration can enhance the creation and operation of DTs for civil infrastructure, providing actionable insights for maintenance and asset management. For example, ref. [13] provides a comprehensive review of how AI, IoT, and BIM converge to drive digital twins in infrastructure management. Ref. [14] propose a conceptual framework that uses multi-sensor data fusion to build digital twin models for smart civil infrastructure. Ref. [15] demonstrates how LiDAR point clouds can be used to generate accurate structural representations for digital twin systems.

In particular, DTs have proven valuable for highway asset management [16] and for developing intelligent decision support systems for road maintenance that integrate sensor data and predictive analytics [17]. Collectively, these studies illustrate the transition from theoretical DT models to practical, AI-enabled systems that enhance the monitoring, management, and sustainability of transportation infrastructure.

A crucial enabler for road DTs is the transformation of survey data into semantically rich information models. While LiDAR and photogrammetry provide high-resolution geometric datasets, raw survey outputs are not directly usable for DTs because they lack semantic structure and interoperability with asset management platforms. To address this, recent research has focused on workflows that convert survey data into BIM models, enabling both geometric accuracy and semantic enrichment. Semi-automated frameworks have been proposed to define Industry Foundation Class (IFC) alignment entities from LiDAR data acquired by MMS [18], while procedural point cloud modeling approaches support both scan-to-BIM and scan-vs-BIM applications [19]. Road detection and parametrization methods that integrate BIM and GIS have been applied to LiDAR datasets to create enriched road models [20]. Other studies have addressed the construction and maintenance of geometric digital twins for roads, emphasizing continuous updates and integration with asset management [21,22]. Approaches for BIM-GIS integration enable intelligent management systems for roads [23], and practical BIM applications for existing road infrastructures demonstrate the feasibility of semantic enrichment for operational use [24]. Finally, systematic reviews of open infrastructure BIM highlight standardized workflows and interoperability strategies critical for the scalability of road DTs [25].

Scan-to-BIM approaches show how point clouds can be segmented, reconstructed, and exported as BIM objects, providing a pathway to generate semantically enriched digital models of road infrastructure. However, many workflows still rely on human supervision and ad hoc rules for identifying and parametrizing road elements. Recent studies illustrate various strategies: fully automatic digital twinning from semantically labeled point cloud data [26], application-oriented frameworks for infrastructure modeling [27], and generation of IFC-compliant road models with semantic attributes [28]. Reviews of scanning technologies emphasize the integration of point clouds into BIM for diverse infrastructure contexts [29], while methodological adaptations have been applied even to complex surfaces such as stone pavements [30].

A summary of representative studies, highlighting the diversity of sensors, scales, and application focuses in road DT research, is presented in Table 1.

Despite these advantages, most existing road DT studies either prioritize large-scale coverage at the expense of geometric and semantic detail, or focus on high-precision modeling of individual assets without addressing network-level integration. As a result, a unified framework capable of combining high-resolution mobile mapping data, municipal geodatabases, and standardized semantic models for urban road networks is still largely missing.

Moreover, several methodological and technological challenges remain. First, there is no widely adopted and road-specific schema within BIM or IFC standards, which limits interoperability and the consistent representation of road assets across platforms. Second, the automation of point cloud segmentation and classification remains limited, with many workflows still relying on manual or semi-automatic procedures that hinder scalability and reproducibility. Third, the alignment of MMS-derived geometry with existing municipal datasets is often problematic due to discrepancies in spatial resolution, positional accuracy, data structure, and coordinate reference systems.

These limitations highlight the need for integrated, scalable, and semantically consistent workflows that can bridge high-resolution survey data and authoritative urban geodatabases within a digital twin framework.

In summary, while DT applications for roads have demonstrated clear benefits for monitoring and predictive maintenance, their adoption in urban contexts remains underdeveloped. At the same time, methodologies for deriving BIM models from survey data are advancing but continue to face challenges of automation, standardization, and integration. Bridging these two strands—the development of scalable DTs for complex city road networks and the establishment of robust BIM-based modeling workflows—represents a critical research direction that this study aims to address.

### 1.3. BIM Dimensions

As urban digital twins increasingly rely on interoperable and standardized information environments, BIM emerges as a key enabler for structuring and managing the complex data ecosystem of road infrastructure. Within this study, BIM serves as the semantic and organizational framework that connects heterogeneous geospatial datasets, supporting the principles of openness and transparency promoted by public digital transformation policies.

Open BIM represents an open and collaborative approach to the design and management of building information, ensuring interoperability across disciplines and software environments. It promotes transparency, accessibility, and long-term usability of digital information, enabling multidisciplinary collaboration throughout the entire asset lifecycle. A core element of the Open BIM approach is the use of open and standardized data models—particularly the IFC—which allow data and models to be exchanged between various software platforms without loss of information. Adopting IFC standards ensures that all stakeholders—from designers and engineers to contractors, operators, and public authorities—can work synergistically, regardless of the proprietary tools used. This interoperability is especially relevant in complex urban contexts and in the management of public infrastructure, where transparent and standardized information exchange is essential for project validation, monitoring, and long-term maintenance. By fostering open data ecosystems, Open BIM supports the transition toward more sustainable, inclusive, and accountable urban management.

Building upon these principles, this study adopts the IFC 4.3 schema [42], the most recent official version of the standard developed by buildingSMART International. IFC 4.3 extends BIM interoperability to horizontal infrastructure, including roads, railways, bridges, ports, and waterways.

Urban road systems are inherently spatially distributed assets, and IFC 4.3 enables their integration within GIS platforms and DT environments, supporting urban mobility simulations, environmental analyses, and coordination with utilities and underground networks. The use of IFC 4.3 ensures compatibility with open standards and facilitates the integration of BIM with GIS data, mobile mapping outputs, and institutional geodatabases, establishing a consistent framework for city-scale interoperability.

In this study, BIM was therefore adopted as the core environment for representing the urban road infrastructure, integrating geometric, semantic, and spatial information within a single parametric model. Each element of the model is geometrically represented and enriched with semantic attributes—such as identifiers, material type, and maintenance status—forming a digital foundation for the urban road digital twin.

In this context, the Open BIM paradigm provides the conceptual and technical basis for harmonizing heterogeneous data sources within a transparent, standards-based workflow aligned with the operational needs of public administrations.

This framework creates a continuous flow of information between design, construction, and territorial management systems, enabling multi-scale analysis, monitoring, and decision-making for urban road networks. In this sense, BIM acts as the semantic backbone of the urban digital twin, allowing real-time updates, multi-source data integration (LiDAR, GIS, geodatabases), and monitoring of infrastructure conditions.

BIM is not limited to 3D geometry but it can incorporate additional dimensions: 4D—Time/project scheduling; 5D—Cost management; 6D—Sustainability/energy performance; 7D—Asset management/lifecycle monitoring. Even if all these dimensions are not implemented in this study, acknowledging them highlights BIM’s evolution and multi-disciplinary potential for urban infrastructure modeling.

In addition to geometric and semantic richness, BIM models are characterized by their Level of Development (LoD), which defines the extent of detail, precision, and reliability of model elements. LoD specifies how much information is included in the model, ranging from coarse representations suitable for conceptual design to highly detailed representations for construction and asset management. According to the BIMForum LoD Specification (2023) [43] and UNI 11337-4:2017 [44], the main LoD categories are:

- LoD100: conceptual representation, approximate size, shape, and location; minimal semantic information.
- LoD200: general layout, approximate geometry, and basic attributes; suitable for planning and coordination.
- LoD300: precise geometry and dimensions; suitable for construction documentation.
- LoD350: includes interfaces and connections with other systems.
- LoD400: detailed fabrication-level geometry with complete specifications, suitable for construction and detailed analysis.
- LoD500: as-built or as-maintained representation, reflecting the actual condition of the built asset.

While ISO 19650-1:2018 [45] and EN 17412-1:2020 [46] introduce the broader concept of *Level of Information Need*, this study adopts the LoD terminology for clarity and comparability with existing BIM practice. For this study, LoD200 and LoD400 were selected to balance coverage and precision in the urban road network:

- LoD200 provides a city-scale overview, derived primarily from GIS layers and supplemented with LiDAR-derived information. Pavements, sidewalks, and vertical assets are represented at a simplified geometric level, enabling integration with other urban datasets. This LoD enables city-scale integration and forms the foundation for multi-source data fusion.
- LoD400 focuses on selected road segments, incorporating high-fidelity reconstruction of vertical assets and detailed pavement geometry derived directly from MMS point clouds. This level supports local-scale advanced monitoring, analysis, and management within the digital twin framework.

Intermediate LoDs such as LoD300 or LoD350 were not used because LoD300 requires highly detailed GIS or survey data not fully available for city-scale coverage and LoD350 focuses on interfaces and connections, which are less relevant for the road DT objectives. Thus combining these two LoDs, the workflow ensures scalable and interoperable modeling, where LoD200 supports broad urban integration and LoD400 allows targeted, high-resolution assessment of critical infrastructure elements. This dual-scale approach ensures a scalable and interoperable digital representation, bridging the gap between traditional 2D GIS datasets and fully parametric 3D infrastructure models.

The adoption of BIM for urban infrastructure is increasingly supported by regulatory frameworks and scientific research. In Europe, the EU Directive 2014/24/EU [47] promotes BIM use for large-scale infrastructure projects to improve interoperability, reduce costs, and enhance lifecycle management. Italy has reinforced this approach through the Italian Public Contracts Code (D.Lgs. 50/2016) [48] within the new Legislative Decree 36/2023 [49] mandating progressive BIM adoption in public works, including roads, bridges, and urban networks, with specified minimum LoDs and digital deliverables depending on project complexity. These regulations highlight BIM’s relevance not only for construction but also for monitoring, managing, and maintaining urban infrastructure.

Beyond regulatory adoption, the evolution of BIM toward DT-oriented frameworks has also been widely explored in the scientific literature, in fact the growing body of research on BIM–DT integration provides the scientific foundation for this study. Over the past decade, BIM has progressively evolved from a construction-centric tool into a multi-disciplinary framework capable of supporting the creation and management of urban digital twins. This transition reflects a growing interest in leveraging BIM as a semantic backbone that integrates heterogeneous data sources—including LiDAR point clouds, GIS layers, and authoritative geodatabases—within interoperable, information-rich environments.

Recent studies have explored BIM–DT integration across various domains. Jiang [50] demonstrated the use of BIM-based digital twins for intelligent building construction management, highlighting the potential of real-time data exchange for process optimization. Similarly, Eneyew et al. [51] and Baghalzadeh Shishehgarkhaneh et al. [52] emphasized the convergence of BIM and the IoT as a foundation for smart infrastructure, where sensor networks enable continuous performance monitoring and predictive maintenance.

Other works have extended BIM–DT frameworks to bridge and infrastructure management. Tita et al. [53] and Honghong et al. [54] demonstrated that combining BIM with DT technologies supports full lifecycle assessment and digital transformation in structural engineering, enhancing decision-making during operation and maintenance phases. Boje et al. [55] outlined research directions toward semantic Construction DTs, stressing the need for standardized data models to enable interoperability among BIM, GIS, and sensor-based systems.

Several systematic reviews have consolidated these advances. Deng et al. [56], Shahzad et al. [57], and Tuhaise et al. [58] identified a clear research trend: the shift from BIM as a static information repository to a dynamic, data-driven digital representation capable of supporting real-time updates, simulation, and lifecycle management. These studies converge on the view that BIM, when enhanced through open standards such as IFC and CityGML, provides the structural and semantic foundation for multi-scale, interoperable DTs in the built environment.

Overall, this body of research confirms that BIM’s evolution toward DT-oriented modeling is not limited to buildings but extends to horizontal and urban infrastructures, including road networks. In this context, BIM acts as a multi-domain integrator, connecting geometric, semantic, and temporal dimensions across diverse datasets. The present study builds upon this trajectory by employing BIM as the central information framework for an urban road DT, exploiting its interoperability (via IFC 4.3) and multi-scale capabilities (through LoD200 and LoD400) to support both city-scale integration and detailed infrastructure assessment. This conceptual and technological background underpins the data integration workflow described in Section 2, where BIM serves as the structural framework through which multi-source geospatial data—LiDAR, GIS, and institutional databases—are harmonized to generate an interoperable digital representation of the urban road infrastructure.

## 2. Materials and Methods

This section describes the datasets, tools, and workflows employed to create a digital twin of the urban road network in Turin. The proposed methodology relies on the integration of geometric, semantic, spatial, and institutional information, combining data acquired through MMS with official municipal GIS layers, authoritative geodatabases, and BIM. The section first introduces the study area, providing the urban and infrastructural context, and then details the data sources, processing workflow, and integration procedures used to generate a multi-source digital representation of the road infrastructure. This framework aims to demonstrate how heterogeneous data can be harmonized to support monitoring, analysis, and management of urban roads within a complex city environment. The proposed integration pipeline should therefore be understood as a DT-ready information environment. Its multi-modal, multi-scale, and multi-level structure ensures that heterogeneous sources are harmonized and semantically aligned, enabling future extensions toward dynamic DT functionalities.

### 2.1. Study Area

**Figure 2:**
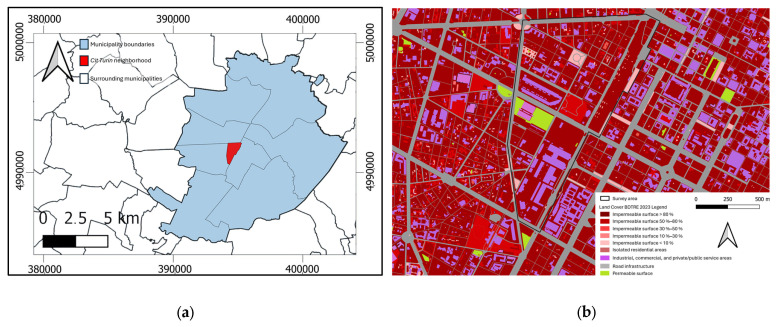
(**a**) Localization of the area of interest: in blue is highlighted the municipality boundaries of Turin (Italy), in red is highlighted the case study area. (**b**) Map of the urban density of the studied area, obtained from the Land Cover Piemonte dataset provided by the Geoportale Piemonte. The map shows the spatial distribution of land use and urban density, providing valuable information on the characteristics of the built environment.

The study was conducted in the city of Turin (Italy), focusing on a representative urban road segment characterized by mixed vehicular and pedestrian circulation. The selected area, presented in Figure 2a, includes a portion of the *Cit Turin* neighborhood, part of the 3rd district, covering approximately 1.15 km^2^. This area presents a variety of pavement conditions, geometric configurations, and surrounding built elements, making it particularly suitable for testing the integration of multi-source spatial data. The study area is projected in the national reference system ETRS89/UTM zone 32N (EPSG:25832), ensuring spatial consistency across all datasets.

The urban fabric is dense and predominantly residential, with a continuous layout where 80% of the surface is impermeable, as shown in Figure 2b, derived from the Land Cover Piemonte dataset provided by Geoportale Piemonte.

**Figure 3:**
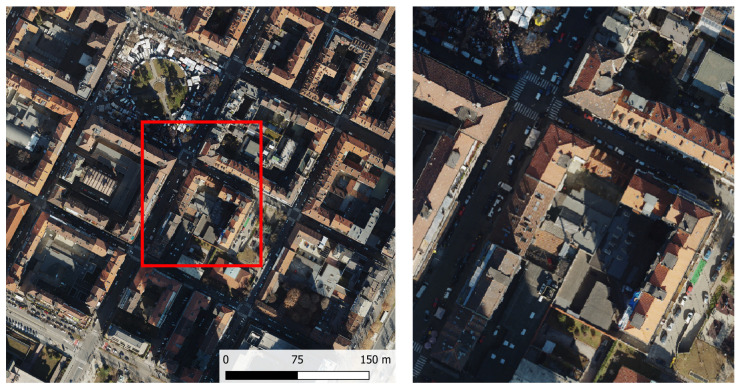
Aerial orthophoto (dated February 2022, flight altitude 1000 m). The red box highlights a zoomed-in area showing a dense urban environment characterized by narrow streets and significant shading caused by closely spaced buildings.

The area encompasses a diverse range of public infrastructure, including the city courthouse, a bus terminal, several metro stations, a public park, and a neighborhood market. An orthophoto obtained from aerial surveys (February 2022, flight altitude 1000 m) illustrates the morphological characteristics of the neighborhood (Figure 3). The dense building pattern and narrow streets produce extensive shaded areas, which limit the effectiveness of aerial imagery acquisition and complicate the accurate identification and monitoring of road and infrastructure conditions. These challenges reinforce the importance of ground-based acquisition systems, such as mobile mapping, and ensure a more complete representation of the urban environment.

The study focuses on the urban road network within the selected area, which includes a mix of arterial, secondary, and local streets, supporting both vehicular and pedestrian circulation. The selected segments were chosen to capture a variety of road geometries, surface types, and urban interactions, providing a suitable testbed for multi-source data integration and DT modeling.

The total analyzed network consists of approximately 40 km of road segments, including arterial roads such as *Corso Vittorio Emanuele II*, *Corso Francia* and *Corso Inghilterra*, secondary roads, and local streets. Road widths vary between 5 and 15 m, with lane configurations ranging from single to multi-lane roads. Sidewalks are present along all segments, while some streets include cycle lanes or bus stops, representing areas with mixed traffic interactions. The road surface conditions within the study area are heterogeneous, with segments exhibiting smooth asphalt, patchwork repairs, and areas with cracks or potholes, providing diverse scenarios for pavement monitoring.

Table 2 summarizes the key characteristics of the analyzed road segments, including type, length, width, number of lanes, surface condition, and special features. These details provide context for the study, enabling the selection of appropriate methods for accurate geometric and semantic modeling of the urban road network.

This detailed characterization allows for tailoring the data acquisition and modeling workflow, ensuring that the DT accurately represents the geometric, semantic, and functional properties of the urban road network.

### 2.2. Data Sources

The proposed framework relies on heterogeneous spatial data obtained from both acquisition campaigns and institutional sources. These datasets were classified into four main categories: Urban Mapping, GIS Data, Authoritative Geodatabase, and BIM Model. This multi-source approach enables the integration of geometric, semantic, and spatial information necessary for building a coherent and interoperable DT of the urban road infrastructure.

**Figure 4:**
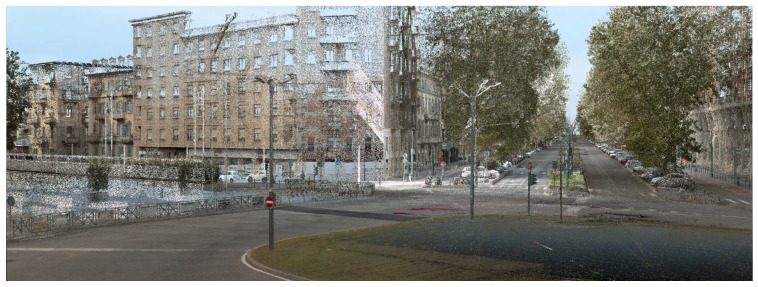
Portion of the LiDAR-derived point cloud.

Urban Mapping: high-resolution LiDAR point clouds were acquired using a MMS mounted on a vehicle. The system integrates LiDAR, GNSS, IMU, and RGB cameras, allowing the generation of dense and accurately georeferenced point clouds of the road surface and its surroundings. These data provide the geometric foundation for the digital model, enabling detailed reconstruction of the road morphology and associated urban elements such as curbs, sidewalks, and street furniture (Figure 4). The MMS survey, conducted in 2023, produced a LiDAR point cloud with an average density of 1500 pts/m^2^ and a range accuracy of ±1 cm. The acquisition was performed at an average vehicle speed of 30 km/h, ensuring full coverage of the carriageway and sidewalks. Under these conditions, the system achieves a nominal absolute accuracy of 1–3 cm and a relative accuracy below 1 cm for short-range measurements (<15 m). The raw data were processed to remove noise and align individual trajectories, resulting in a georeferenced and colorized point cloud suitable for subsequent modeling.

A known limitation of MMS in dense urban environments concerns occlusions generated by parked vehicles, vegetation, temporary structures, and street furniture, which may locally reduce point density or prevent full sampling of certain assets. Such effects impact the completeness of the dataset used in the manual and semi-automatic pre-filtering stages but are inherent to MMS acquisition rather than to the proposed workflow.

GIS Data: the municipal GIS database of Turin was employed to provide essential spatial layers, including road centerlines, administrative boundaries, and land-use zones. In particular, the Carta Tecnica Comunale (CTC)—Turin’s official large-scale topographic map (latest update September 2024)—was used as a base layer for spatial referencing. The CTC provides high-accuracy vector data describing buildings, road layouts, and urban features at a detailed scale (1:1000). These datasets supported the alignment of the BIM model within the broader urban context and facilitated the integration of multi-scale spatial information.

Authoritative Geodatabase: a distinction is made between GIS data and the Authoritative Geodatabase, as they serve complementary but distinct purposes. While GIS data primarily describe the spatial geometry and topology of the urban context, the Authoritative Geodatabase represents the institutional and semantic component of the information environment. Furthermore, official municipal geodatabase supplied validated and standardized information regarding road classification, ownership, surface material, and maintenance status. These institutional datasets were linked to the modeled BIM elements to ensure semantic consistency with official records and enhance the reliability and traceability of the digital representation.

BIM Data: the BIM environment was employed to construct a parametric representation of the road infrastructure, translating geometric information derived from the MMS point clouds into semantically enriched objects. Each element (e.g., pavement surface, sidewalk, median) was assigned attributes from the authoritative geodatabase and GIS layers, forming an information-rich model that supports further analysis, monitoring, and management within the digital twin framework.

Parametric representations were generated at two levels of detail, each addressing specific analytical and integration purposes.

- The first model (LoD200) was created primarily from municipal GIS layers and supplemented with information derived from the LiDAR point cloud, particularly regarding pavement geometry and surface condition. It also included a simplified representation of key road assets, such as vertical poles and street furniture, modeled at a lower geometric resolution. This model provided a generalized yet information-rich overview of the entire study area, supporting integration and visualization within the GIS environment.
- The second model (LoD400) focused on selected road segments and incorporated detailed geometric features extracted directly from the MMS data. Vertical assets were reconstructed with higher geometric fidelity, while semantic attributes from the authoritative geodatabase—such as surface material, ownership, and maintenance status—were integrated to support advanced analyses and infrastructure management.

This dual-scale modeling approach ensured interoperability between datasets of varying resolution, enabling both city-scale representation and detailed local assessment within the digital twin framework.

### 2.3. Data Integration Workflow

This study follows a system-oriented research design aimed at developing and validating an integrated workflow for urban road DT construction. Rather than benchmarking individual algorithms under controlled conditions, the proposed methodology focuses on combining heterogeneous data sources—mobile mapping LiDAR, municipal GIS layers, and authoritative geodatabases—into a coherent, interoperable, and semantically enriched BIM-based DT framework. The workflow is evaluated at the system level, focusing on data integration consistency, semantic completeness, and geometric accuracy, rather than on isolated algorithmic performance.

Building on the multi-source datasets described in Section 2.2, a structured data integration workflow was implemented to generate a coherent and interoperable DT of the urban road infrastructure. The workflow was designed to combine geometric information from LiDAR point clouds, spatial data from GIS layers, semantic and institutional attributes from the municipal geodatabase, and parametric representations in BIM, enabling both city-scale visualization and detailed local analysis. The method focuses on generating an interoperable, multi-modal digital representation of the urban road network. While the resulting model is designed to be compatible with downstream DT implementations, the present study does not include dynamic data streams, system APIs, or bi-directional communication mechanisms. Instead, the emphasis is placed on establishing a semantic and geometric foundation suitable for integration within future DT platforms. The resulting BIM-based DT provides a static but interoperable representation of the urban road network. Future integration with IoT sensor networks, connected vehicle data, and real-time traffic monitoring could transform the framework into a dynamic digital twin, enabling predictive maintenance and continuous condition assessment.

This approach ensures that all datasets—acquired through ground-based and institutional sources—are spatially aligned, semantically consistent, and geometrically accurate, forming the foundation for subsequent modeling, monitoring, and management tasks. The following subsections describe the main steps of the workflow, including point cloud processing, BIM, GIS and geodatabase integration, and model interoperability, with the software tools employed at each stage. The main steps are described as follows:

Point cloud processing and Feature extraction: the LiDAR point clouds acquired via MMS were processed to extract relevant geometric and semantic information for the DT. The workflow included preprocessing, classification, and feature extraction, ensuring that both pavement and urban assets were accurately represented. In the preprocessing stage the raw point clouds were first cleaned to remove noise and spurious points caused by sensor artifacts or moving objects. Individual trajectories were aligned and merged to produce a georeferenced, continuous 3D representation of the road network and its surroundings. Then the resulting point cloud was classified into key categories, including ground/pavement, buildings, vegetation, wire, static and dynamic car, and vertical assets such as poles, signage, and street furniture. Point cloud classification was guided by geometric and radiometric criteria commonly adopted in urban MMS processing, including height above ground, local surface normal orientation, point density, and neighborhood variance. Automated classification results were visually inspected and manually corrected in areas affected by occlusions or sparse sampling, particularly in narrow streets and shaded zones. Then the vertical assets were identified and segmented through clustering techniques based on geometric properties (height, verticality, and cross-sectional dimensions). Density-based spatial clustering (DBSCAN) was adopted for asset segmentation due to its ability to identify objects of arbitrary shape and to handle noise without requiring a predefined number of clusters. Clustering was performed using spatial proximity and vertical continuity constraints, following practices commonly reported in mobile LiDAR-based urban asset extraction [59,60].

Each cluster corresponding to a road asset was assigned a unique identifier, and its position and geometry were exported for integration with the BIM model. Pavement condition indicators, derived from local surface irregularities detected in the point cloud, were also associated with the corresponding road segments.

The classified and segmented point cloud served as a foundation for the creation of the BIM models at different levels of detail. In the LoD200 model, vertical assets and pavement features were represented at a simplified geometric resolution, while in the LoD400 model, selected segments and assets were reconstructed with higher geometric fidelity. Semantic attributes from the authoritative geodatabase and GIS layers were linked to the parametric objects, producing an information-rich representation of the urban road infrastructure suitable for analysis, monitoring, and management within the digital twin framework.

BIM: parametric BIM models were developed using Autodesk Revit 2025 with Dynamo and FreeCAD with Python 3.12 scripts. Each road segment, pavement, sidewalk, and vertical asset was modeled as a parametric object with associated semantic attributes, two complementary model were created: the LoD200 model generated from GIS layers and supplemented with LiDAR-derived pavement and asset information and LoD400 model focused on selected segments and included higher-fidelity reconstruction of vertical assets and detailed pavement geometry. Automation via Dynamo routines and Python scripts ensured consistency across segments and maintained hierarchical relationships between pavement, sidewalks, and vertical assets. The integrated BIM model was exported in IFC format to ensure interoperability and potential use within urban-scale digital twin environments. The separation between LoD200 and LoD400 models allows the workflow to balance scalability and detail, ensuring that city-wide coverage does not compromise the accuracy required for local asset-level analysis.

GIS and Authoritative Data Integration: semantic attributes from the municipal geodatabase—such as road classification, surface type, and maintenance status—were linked to BIM objects using Python and Dynamo environments. This step guaranteed that each modeled element corresponded to an officially recognized entity and enhanced data reliability. Attribute consistency was verified by cross-checking unique identifiers, spatial correspondence, and classification codes between BIM objects and the authoritative geodatabase.

The workflow described above was implemented using a combination of specialized software and programming tools selected for their specific capabilities and compatibility with open standards. CloudCompare was employed for point cloud segmentation, classification, and meshing, providing accurate geometric representations of pavements and vertical assets. Autodesk Revit 2025 was chosen for LoD400 BIM because of its advanced parametric design capabilities, enabling detailed reconstruction of selected road segments and high-fidelity modeling of vertical assets. For LoD200, FreeCAD was used due to its ability to easily handle geographic coordinates and integrate GIS-derived data, allowing the generation of a generalized yet information-rich model covering the entire study area. Dynamo scripts were used within Revit to automate repetitive modeling and attribute assignment tasks, ensuring hierarchical consistency among pavement, sidewalks, and assets. Python scripts were employed for multiple purposes: clustering and centroid extraction of vertical assets from the point cloud, and automating the 3D modeling workflow in FreeCAD, allowing all elements to be generated simultaneously while preserving semantic attributes.

All tools were configured to ensure compatibility with open standards (e.g., IFC), facilitating interoperability and smooth data exchange between BIM and GIS domains. The combination of these tools allowed the workflow to be both efficient and scalable, supporting multi-scale modeling and integration of heterogeneous datasets within the digital twin framework.

While current workflows rely on Python scripts and Dynamo routines for processing and integrating multi-source data, AI and machine learning methods could further enhance automation, semantic enrichment, and predictive capabilities. AI models could facilitate automated classification of urban assets, anomaly detection across heterogeneous datasets, simulation of traffic and pedestrian flows, and optimization of infrastructure management strategies. Integrating these AI capabilities would enable the transition from a static, DT-ready environment to a dynamic, data-driven urban road DT. These AI-related extensions are not implemented in the present study and are discussed as prospective enhancements enabled by the structured, semantically enriched datasets produced by the workflow.

### 2.4. Methodological Framework

**Figure 5:**
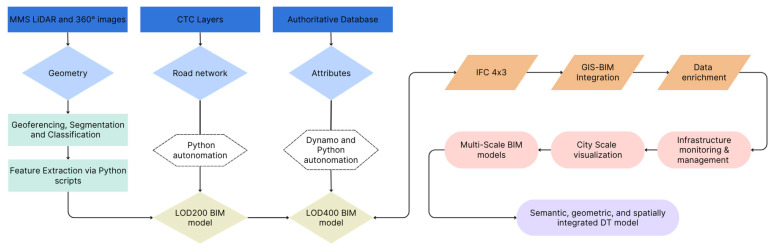
Methodological framework for the integration of multi-source geospatial data within the BIM–GIS workflow. The flowchart is organized into six layers: Input layers (blue), pre-processing layer (green), modeling layer (yellow), integration layer (orange), output layer (pink) and Final DT layer (purple).

The methodological framework adopted in this study is illustrated in Figure 5, which summarizes the overall data flow, integration, and modeling strategy. The framework is designed to transform heterogeneous geospatial datasets into an interoperable, multi-scale digital twin of the urban road infrastructure. It builds upon four core components that interact through open data standards and semantic interoperability:

- Urban Mapping provides the geometric representation of the real-world environment through LiDAR point clouds and 360° images.
- GIS defines the spatial framework for data alignment, referencing, and visualization at the territorial scale.
- BIM structures, stores, and manages the geometric and semantic information in a parametric, multi-scale environment.
- Authoritative Geodatabases ensure institutional reliability, providing standardized attributes (e.g., surface type, administrative boundaries, maintenance zones).

Together, these components form a multi-source integration pipeline that supports monitoring, maintenance, and data-driven urban management. Moreover, this layered structure ensures traceability of information from raw sensor data to semantically enriched DT-ready outputs.

The workflow integrates both automated and semi-automated procedures. Custom Python scripts were developed for clustering and centroid extraction from LiDAR data and for automating BIM tasks within the FreeCAD environment. Dynamo was used to optimize the parametric modeling process in Revit for higher LoD representations.

The framework employs two BIM LoDs—200 and 400—to balance scalability and precision (Table 3).

- LoD200 models were generated from GIS layers and LiDAR-derived information, supporting city-scale integration and general visualization.
- LoD400 models were developed for selected road segments, ensuring detailed reconstruction of pavements and vertical assets for local-scale monitoring and management.

This dual-level modeling approach enables both broad urban integration and high-resolution analysis, bridging the gap between 2D GIS datasets and fully parametric 3D infrastructure models. This methodological framework ensures reproducibility and scalability, enabling the extension of the workflow to other urban contexts and infrastructure typologies within a digital twin perspective. The dual LoD approach not only balances scalability and precision but also provides AI-ready datasets. LoD200 models facilitate city-scale analyses, while LoD400 segments supply high-fidelity geometry and semantic attributes suitable for training predictive models for pavement condition, asset monitoring, and maintenance planning.

The total duration of the proposed workflow depends on dataset size, point cloud density, and the amount of manual intervention required. For the presented case study, covering approximately 45 km of urban roads with over 2000 vertical elements (including streetlights, traffic lights, and vertical signs) and extensive horizontal markings, the workflow required roughly ~15 days (Table 4) in total. This estimate excludes the time spent testing and refining scripts for clustering and BIM. This includes MMS acquisition, point cloud pre-processing, segmentation and clustering of vertical elements, extraction of horizontal markings, and BIM parametric modeling. While the current workflow is feasible for medium-scale networks, further automation and optimization, particularly in clustering and BIM generation, are recommended to scale the approach for entire city-wide networks.

These values provide a first-order estimate of workflow efficiency and serve as a reference for future optimization and comparative evaluation.

## 3. Results

The results demonstrate the effectiveness of the proposed approach in integrating road assets into the broader DT framework of the city. This methodology highlights the strategic use of geospatial data acquired through MMS for representing and managing urban road infrastructure in complex city contexts. This section is structured in alignment with the primary stages of the developed workflow. Specifically these results demonstrate not only the technical feasibility of the workflow, but also its scalability, interoperability, and readiness for operational deployment within municipal digital twin environments.

### 3.1. Point Cloud Segmentation and Feature Extraction

The first stage of the workflow involved the processing and segmentation of the MMS LiDAR point cloud to isolate the main components of the urban road environment.

A classified point cloud was used to extract both vertical and horizontal road elements, which constitute the geometric foundation for subsequent modeling and data integration.

**Figure 6:**
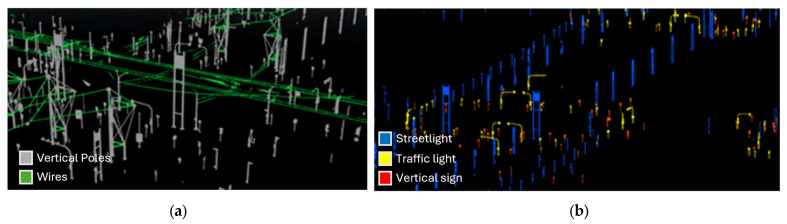
Vertical road asset segmentation step. (**a**) The image show the initial vertical poles class (grey). (**b**) The image show the further subdivision of the vertical poles class into 3 main sub-classes: blue—streetlight; yellow—traffic light; red—vertical sign.

Vertical elements such as poles, streetlights, traffic lights, and vertical signs were derived from the vertical poles class using CloudCompare. Since no pre-trained AI model was available or sufficiently adapted to the specific characteristics of the dataset, a manual classification approach was adopted. Through the application of intensity filters in CloudCompare, it was possible to discretize the vertical pole class into meaningful subcategories based on their reflectance values (Figure 6).

To isolate individual objects, a density-based clustering algorithm was employed. DBSCAN was chosen for its ability to detect clusters of arbitrary shape and effectively separate dense object groups from sparse background noise without requiring a predefined number of clusters. The algorithm relies on two parameters: (i) *ε* (epsilon), which defines the neighbourhood radius, and (ii) *minPts*, the minimum number of points required to form a dense region. Points located within *ε* distance of each other and satisfying the *minPts* threshold are aggregated into clusters, while isolated points are labelled as noise. This behaviour makes DBSCAN particularly suitable for the detection of compact and spatially coherent objects in urban road networks—such as streetlights, traffic signals, or vertical signage—where elements are typically arranged in linear sequences along road axes but must be distinguished from nearby artifacts and spatial noise [59]. Recent studies have revisited and formalized its properties, providing guidance for parameter selection and performance in high-dimensional or irregular-density datasets [60].

DBSCAN has been extensively adopted in mobile mapping, LiDAR, and road-infrastructure applications, where it serves as a preliminary segmentation and filtering step. Examples include ground and curb detection in MLS data, object extraction from urban point clouds, and noise removal prior to semantic classification [61,62,63,64]. Several works have also extended DBSCAN for datasets with variable density—typical of MMS acquisitions—through adaptive *ε* estimation or hierarchical density clustering [65].

In this implementation, DBSCAN is applied directly to the segmented MMS point cloud of the infrastructure element of interest. After extracting the class of points corresponding to streetlights, the algorithm is executed as follows:

- Input: LiDAR point cloud
- Output: cluster centroids

1. Load point cloud and extract XYZ coordinates.
2. Apply DBSCAN with parameters (*ε*, *minPts*) to identify point clusters.
3. For each cluster (excluding noise):
  - compute the centroid as the mean of its XYZ coordinates.
4. Store all centroids in a table and export them to CSV.

The chosen values (*ε* = 5 m, *minPts* = 5) were determined empirically through iterative testing to account for the typical spatial footprint of a lamp post in the MMS acquisition geometry. Points labelled as noise (*label* = −1) are automatically excluded, while each remaining label corresponds to an individual infrastructure object. Once clusters are identified, the centroid of each object is computed as the mean of its XY Z coordinates, which provides a geometrically consistent representative point for BIM object placement.

The resulting centroids are exported to a CSV file and used as anchor points for generating parametric BIM families.

The extracted features were then exported as structured attribute tables and spatial point geometries, which served as inputs for the BIM phase. In this way a total of 1178 streetlights, 932 vertical signs and 236 traffic lights were extracted. This result confirms that the proposed clustering strategy is effective for large-scale asset inventory generation from MMS data, even in the absence of pre-existing authoritative records.

It is important to note that DBSCAN was used in this study primarily as a screening and clustering tool to isolate candidate infrastructure elements, rather than as a full object-detection pipeline requiring quantitative validation. The extracted elements, such as streetlights, traffic lights, and certain vertical signs, were not present in any authoritative municipal database, and no reliable ground truth dataset was available for the study area. Consequently, standard performance metrics such as false positives, false negatives, precision, or recall could not be computed. Instead, the extracted clusters were directly used to support the subsequent BIM phase, which constitutes the primary objective of the workflow. Visual inspection and spatial consistency checks against high-resolution orthophotos and MMS imagery confirmed that the majority of extracted clusters correspond to real-world assets, with negligible spatial misalignment along road corridors.

**Figure 7:**
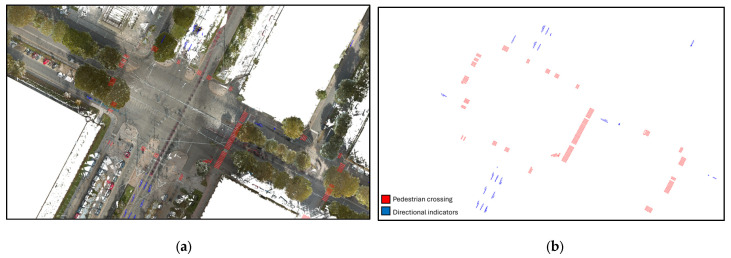
Example of horizontal signage detection: blue vectors represent directional indicators, while red vectors highlight pedestrian crossings. (**a**) The overlay of vectors on the point cloud with RGB values, demonstrating their spatial alignment. (**b**) The vector extraction, highlighting discrepancies in geometry due to low signal reflectivity. This issue corresponds to the poor physical condition of the element, reflecting its degraded state in reality.

The ground-class points of the LiDAR dataset were analyzed to identify and extract horizontal road markings, with a particular focus on crosswalks. Leveraging the higher reflectivity of pavement markings, a custom Python-based workflow was applied to isolate these points from the surrounding road surface. The filtered points were then clustered to separate individual markings, and their geometries were extracted using Shapely. The resulting polygons and lines were stored as vector reference layers in GeoPandas, providing a consistent and reproducible representation of road markings. These layers were subsequently used to guide the parametric modeling of the pavement within the BIM environment, ensuring geometric alignment between the LiDAR-derived measurements and the modeled surface (Figure 7). The algorithm is executed as follows:

Input: LiDAR point cloud (ground-class points)
Output: Vector layers representing horizontal road markings

1. Select ground-class points from the LiDAR dataset.
2. Use intensity values to isolate high-reflectivity points corresponding to road markings.
3. Apply clustering to separate individual marking elements (e.g., crosswalk strips).
4. For each cluster:
  - reconstruct marking geometry using geometric fitting tools;
  - generate polygon.
5. Store all extracted geometries as vector layers for mapping and analysis.

Notably, geometric discontinuities observed in the extracted vectors correlate with areas of reduced reflectivity in the point cloud, which in turn reflect the degraded physical condition of the markings. This demonstrates the potential of MMS-derived data not only for geometric reconstruction but also for indirect condition assessment.

**Figure 8:**
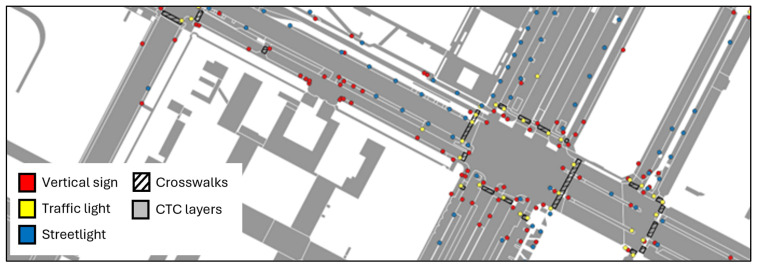
Integration of features extracted from the point cloud into the municipal geodatabase. Colors indicate asset types: red—vertical signs; yellow—traffic lights; blue—streetlights; 5° slanted line hatch—crosswalks; grey—CTC layers.

The resulting classified cloud provided both geometric and semantic separation of infrastructural elements, establishing the geometric foundation for BIM–GIS integration. Moreover, this phase produced a labeled dataset of both vertical and horizontal road features, constituting a valuable resource for the development of future machine learning models aimed at automating and scaling the classification process at the city-wide level. Furthermore, this step also enhanced the municipal geodatabase by incorporating this new information as highlighted in Figure 8.

### 3.2. BIM at Multiple Levels of Development

The classified and enriched point cloud data were used to generate BIM models at two levels: LoD200 for city-scale representation and LoD400 for detailed reconstruction of selected segments. The dual-scale modeling strategy proved effective in balancing computational efficiency and information richness, enabling city-wide coverage without sacrificing the accuracy required for asset-level analysis.

At LoD200, the geometric representation of approximately 45 km of the urban road network was derived by integrating GIS layers—such as the municipal cartography and CTC datasets—with LiDAR-derived features. Custom Python scripts automated geometry generation and attribute mapping within FreeCAD, preserving the EPSG:25832 reference system. Each horizontal element such as road segment, cycling, sidewalk, median, crosswalk and vertical asset was extruded from vector footprints and enriched with attributes including material type, functional class, and maintenance zone. Additionally, medium-scale urban features, such as buildings, were incorporated to improve spatial context and interoperability with the municipal digital twin environment. These entities were also connected to institutional identifiers and thematic layers, ensuring semantic consistency across datasets.

The following pseudocode examples summarize the main workflow steps, with the first example addressing a road section and the second addressing vertical assets.

Input: shapefile

Output: BIM slabs representing selected road artifacts

1. Load shapefile
2. Filter features by attribute TYPE
3. For each polygon feature:
  - read geometry and attribute values;
  - set base elevation and extrusion height according to TYPE;
  - generate a polygonal wire from coordinates;
  - create a face and extrude it into a solid volume;
  - convert the solid into a BIM Structure (IfcType = Road);
  - attach selected attributes as custom properties.

Input: shapefile, with vertical asset locations and type attribute, and BIM element

Output: BIM elements representing the vertical asset model

1. Load shapefile and read features.
2. For each feature:
  - extract type and (x, y) coordinate;
  - import the BIM element;
  - place the elements at the feature’s (x, y) location.
3. Recompute the BIM document to finalize geometry placement.

**Figure 9:**
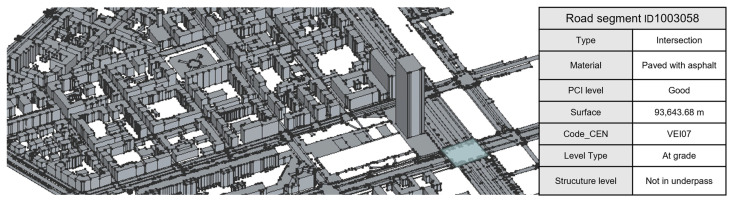
View of a portion of the LoD200 model highlighting the same intersection shown in the high-detail model. On the right, a table of the highlighted intersection, that includes the attributes derived from the municipal geodatabase, is displayed.

The resulting LoD200 model (Figure 9) provides a simplified yet information-rich representation suitable for city-scale analyses, facilitating integration with other municipal GIS datasets and supporting thematic visualization, spatial querying, and data-driven maintenance planning.

For selected critical road segments, a high-fidelity representation was developed at LoD400 following a Scan-to-BIM approach. Segmented MMS point clouds were imported into Revit 2025, where Dynamo scripts were implemented to automatically assign semantic information, link objects to their attributes, and ensure consistency with the LoD200 model and GIS reference data.

**Figure 10:**
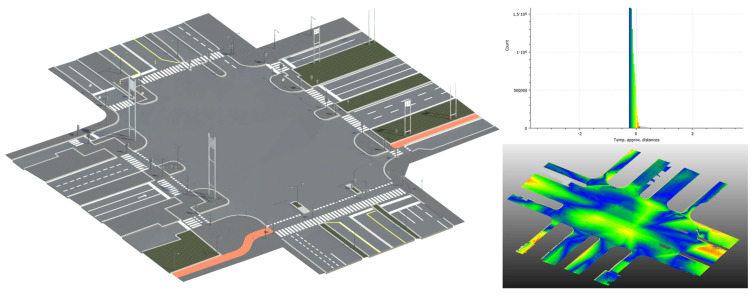
Example of the high-resolution LoD400 model. A Comparison between the high-resolution LoD400 model and the corresponding LiDAR segment is highlighted on the right panel illustrating a deviation analysis, showing a maximum geometric discrepancy of ±3 cm between the modeled and surveyed data.

The modeling strategy distinguishes between pavement reconstruction and vertical asset modeling. Pavement geometry was obtained using a dedicated workflow developed in a previous study by the authors [66], where MMS-derived point clouds are processed to extract continuous road surfaces and convert them into parametric BIM elements. The resulting models (Figure 10) achieved centimeter-level accuracy, validated through cloud-to-mesh deviation analysis (RMSE ± 3 cm) performed on CloudCompare. This level of precision enables condition assessment, deformation tracking, and as-built verification for infrastructure management. This level of accuracy is compatible with requirements for as-built verification, deformation monitoring, and pavement condition assessment in urban environments.

Vertical assets—including lamp posts, traffic lights and vertical signs—were derived entirely from the MMS point cloud. After manual and semi-automatic filtering of the “vertical pole” class in CloudCompare, DBSCAN was applied to separate individual objects. For each cluster, centroid coordinates and bounding geometries were computed, providing both the spatial location and the geometric parameters of the asset. These centroids served as placement points within the BIM environment, where Dynamo scripts imported the coordinates.

To standardize the modeling of vertical infrastructure, an abacus of vertical elements was developed in Revit 2025. This abacus consisted of parametric families representing key urban assets such as streetlights, traffic signs, and vertical sign. Each family was designed to be fully parametric, allowing adjustment of key geometric attributes (e.g., height, diameter, spacing, number of lights) while maintaining semantic consistency.

The abacus was applied consistently across both models:

- At LoD200, simplified versions of the families were used to provide a city-scale representation of vertical assets, ensuring efficient visualization, spatial querying, and thematic mapping.
- At LoD400, the same families were instantiated with higher geometric fidelity, capturing detailed features necessary for condition assessment and deformation analysis.

**Figure 11:**
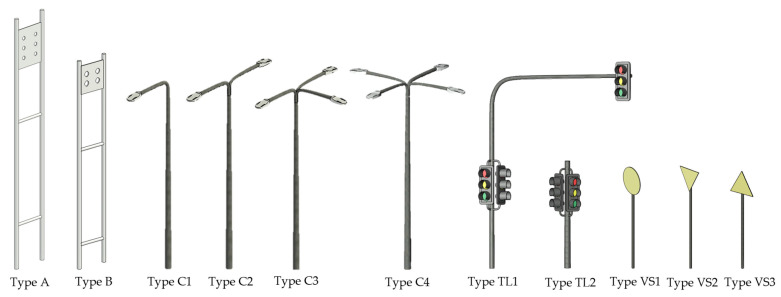
Abacus of vertical element families. Types A, B, C1, C2, C3, and C4 correspond to streetlight elements; types TL1 and TL2 correspond to traffic light elements; and types VS1, VS2, and VS3 correspond to vertical sign elements. Specifically, VS1 represents mandatory and prohibitory road signs, VS2 corresponds to right-of-way signs, and VS3 refers to warning signs.

Using the abacus ensured uniformity in object classification, attribute assignment, and spatial referencing across all BIM models. Additionally, the parametric nature of the families allowed automated population of semantic information and links to geospatial datasets through Dynamo scripts, enhancing the interoperability of BIM and GIS environments. Figure 11 illustrates examples of the vertical element families used in the abacus.

The proposed methodology follows a multi-modal, multi-scale, and multi-level framework.

- Multi-modal refers to the integration of point clouds, imagery, and vector data, leveraging the strengths of each data source to extract geometric and semantic information.
- Multi-scale highlights the ability to process and represent data at different resolutions, from city-scale LoD200 models suitable for urban-scale analyses to high-resolution LoD400 models for detailed asset assessment.
- Multi-level indicates modeling across different scopes: from a generalized city-wide representation of the entire road network, to asset-level detail capturing parametric BIM elements for individual road segments and specific components, including road markings, traffic signs, lamp posts, and traffic lights.

This framework ensures both broad integration across urban areas and fine-grained, asset-specific analysis, supporting applications ranging from visualization and spatial querying to condition assessment and predictive maintenance.

### 3.3. Data Integration and Enrichment

Following geometric modeling, all BIM elements were integrated using the IFC 4.3 schema (Table 5), ensuring compliance with open standards for horizontal infrastructure. This schema facilitated the mapping of key BIM entities—such as IfcRoad, IfcPavement, and IfcSign—and their linkage to external GIS datasets and authoritative municipal geodatabases. By establishing these connections, the workflow transformed raw geometric models into semantically rich, institutionally reliable representations of the urban road network. The use of IFC ensures interoperability across software platforms, avoids vendor lock-in, and supports transparency and reproducibility in municipal infrastructure management.

Spatial alignment across datasets was maintained using the shared coordinate system (EPSG:25832), enabling seamless interoperability between BIM, GIS, and external data sources. In the LoD200 city-scale model generated in FreeCAD, full coordinate reference system (CRS) management preserved precise geographic coordinates, ensuring accurate integration with municipal GIS layers and external geodatabases. For LoD400 high-detail segments modeled in Revit, coordinates were localized to simplify handling and improve software performance. While this approach slightly reduces georeferencing precision, it maintains geometric accuracy at the asset level, facilitating detailed inspection and condition assessment without compromising workflow efficiency.

The integration process also enriched each BIM element with semantic attributes imported from the municipal geodatabase, including surface type, material, functional classification and maintenance. Linking these attributes to IFC entities ensures institutional reliability and enables public administrators to query, filter, and analyze assets in a structured and reproducible manner. For example, maintenance managers can extract all crosswalks within a specific district, identify traffic lights exceeding a given age, or generate reports of streetlights requiring inspection.

Furthermore, the hierarchical structure created by integrating BIM geometry, GIS topology, and tabular data allows the model to function as a semantically consistent and spatially accurate representation of urban roads. This structure supports interoperability with other digital twin layers, such as environmental monitoring, traffic management, and asset lifecycle databases. By combining geometric fidelity, semantic richness, and standard-compliant interoperability, the integrated model provides municipalities with a robust tool for data-driven decision-making, operational management, and long-term planning of road infrastructure.

As a result, BIM entities can be queried, filtered, and aggregated using both geometric and administrative criteria, enabling cross-domain analyses that are not possible using isolated GIS or point cloud datasets alone.

### 3.4. Final Output and DT Readiness

The final outcome of the workflow is a multi-source, interoperable 3D model of the urban road infrastructure, integrating both LoD200 and LoD400 representations within a unified IFC-based environment. The model can be exported in open formats (IFC), ensuring compatibility with visualization tools, analysis platforms, and urban DT frameworks. By combining multi-scale, multi-level, and multi-modal data, this final product establishes a robust foundation for the management and continuous monitoring of urban road networks.

To evaluate the contribution of each data source, a comparative assessment of information content before and after integration was performed (Table 6). The MMS-derived LiDAR data provide high-resolution geometric detail suitable for surface analysis but lack semantic or administrative context. Conversely, municipal GIS and authoritative geodatabases provide rich semantic information—such as road hierarchy, ownership, and maintenance records—but only in 2D form. The integrated BIM-DT combines both, enriching each geometric element with semantic attributes and enabling machine-readable, queryable entities. Table 6 provides a comparative evaluation of the information content and operational readiness of the individual data sources versus the integrated BIM-based digital twin.

At the city scale, the LoD200 model provides an efficient yet information-rich representation of approximately 45 km of urban roads, integrating GIS layers, municipal cartography, and LiDAR-derived assets. This allows municipalities to perform broad analyses such as network-level maintenance planning, traffic safety assessment, and prioritization of interventions. For example, crosswalks, cycling lanes, sidewalks, and street lighting are fully mapped and semantically enriched, supporting data-driven operational decisions. The LoD400 segments provide detailed, asset-level models suitable for condition assessment, deformation tracking, and verification of as-built conditions. High geometric accuracy (RMSE ± 3 cm) ensures reliability for inspection and maintenance tasks.

All BIM elements are semantically annotated and linked to authoritative municipal geodatabases, enabling a variety of public administration applications. These include:

- Condition assessment and monitoring: high-fidelity geometry and semantic attributes enable the accurate evaluation of road assets, including streetlights, traffic signs, and pavement markings, allowing timely identification of maintenance needs.
- Data-driven maintenance planning: by linking each asset to institutional geodatabases, the model provides public administrators with a comprehensive tool for prioritizing interventions, allocating resources efficiently, and documenting maintenance activities.
- AI-readiness and predictive analytics: The structured, machine-interpretable format facilitates the development of predictive models for pavement degradation, asset failure, or traffic sign obsolescence. These models can leverage the labeled dataset produced from point cloud segmentation and BIM parameterization for future automation and scalability.

The integration results in semantically enriched BIM entities, which now combine MMS-derived geometric properties with GIS-derived administrative attributes. These attributes are stored within the IFC 4.3 schema and can be directly accessed by analytical or AI algorithms, transforming raw point clouds into labeled, interoperable datasets.

The multi-level, multi-scale approach bridges the gap between traditional 2D GIS mapping and fully parametric 3D infrastructure modeling, allowing municipalities to seamlessly transition from high-level planning to detailed asset management. The model supports scenario simulations, incremental updates from new surveys, and integration with other urban DT datasets such as building footprints, traffic flow information, or environmental monitoring layers.

Overall, the integration demonstrably enhances both the information content and functional usability of the model. Ultimately, the workflow produces a practical, interoperable DT framework tailored to municipal management needs. It enables continuous, data-driven oversight of the urban road network while providing a platform for future AI-driven applications, predictive maintenance, and policy-informed decision-making. From an operational perspective, this enrichment supports AI-readiness by providing structured, labeled datasets suitable for training predictive models. For instance, linking pavement surface irregularities (from MMS) with road hierarchy and maintenance frequency (from the municipal geodatabase) allows the prioritization of resurfacing tasks based on both physical condition and functional importance. While full AI testing lies beyond the present scope, this data fusion already forms a machine-interpretable foundation for future models in automated defect detection, deterioration forecasting, and maintenance optimization.

This final output represents a key step toward operationalizing city-scale DTs that are both geometrically precise and semantically rich, laying the groundwork for sustainable and efficient urban infrastructure governance. These results demonstrate that the proposed workflow effectively bridges the gap between raw MMS data acquisition and operational DT deployment at the city scale.

### 3.5. Operational Deliverables for Monitoring and Maintenance

The proposed workflow produces several actionable deliverables that directly support urban road monitoring and maintenance. First, the automated segmentation pipeline extracted 1178 streetlights, 932 traffic signs, and 236 traffic-light poles, each represented as an IFC object enriched with geometric attributes and location. These elements constitute a structured and machine-readable asset inventory, fully compatible with municipal GIS datasets and BIM environments.

Second, the reconstructed pavement surfaces enable the derivation of local geometric indicators supporting condition assessment and surface degradation analysis, forming a quantitative basis for monitoring pavement performance across the urban network.

Third, the integration of authoritative municipal databases ensures that each object in the BIM–GIS environment carries associated lifecycle attributes such as installation year, ownership, material type, and maintenance status. This interoperability enables the generation of a unified road asset repository, updated with both pre-existing administrative data and new LiDAR-derived information, significantly enriching the municipal geodatabase.

Beyond monitoring, the BIM models produced through this workflow serve as reliable as-built representations of street assets and pavement geometry. These models can be used to support urban design, restoration of public spaces, and infrastructure planning, enabling municipalities to simulate interventions, evaluate impacts, and coordinate maintenance operations with greater accuracy.

Furthermore, the structured datasets produced by the segmentation and classification pipeline are immediately suitable for AI-based applications. Each vertical and horizontal asset contains geometric parameters (e.g., height, diameter), precise spatial coordinates, and semantic attributes imported from municipal datasets. These machine-readable datasets enable a wide range of predictive analyses, including automated pavement defect detection, deterioration forecasting, visibility analysis, asset recognition, and maintenance prioritization. By ensuring attribute consistency through the IFC 4.3 schema and linking objects to authoritative geodatabases, the workflow supports scalable AI model training and deployment across the entire road network. Based on the structured and semantically enriched datasets produced, several AI-driven applications become immediately feasible, as summarized in Table 7.

## 4. Discussion

The proposed BIM-based DT framework demonstrates the feasibility and effectiveness of integrating heterogeneous spatial datasets for urban road infrastructure management. By combining MMS-derived LiDAR point clouds, municipal GIS layers, and authoritative geodatabases, the workflow successfully bridges geometric precision with semantic richness. The multi-scale structure based on LoD200 and LoD400 models ensures both scalability and flexibility, enabling city-level integration as well as detailed analysis of specific assets. The semantic enrichment through the IFC 4.3 schema provides a transparent, interoperable, and future-proof data environment, supporting long-term integration within DT platforms.

From a technical standpoint, the workflow achieves high geometric accuracy (RMSE ± 3 cm) and maintains full geospatial consistency through the adoption of the EPSG:25832 reference system. The achieved geometric accuracy demonstrates that MMS-based acquisition, when integrated with BIM–GIS workflows, can meet the precision requirements of urban asset management, bridging the gap between survey-grade data and operational DT needs.

Its compatibility with open standards (IFC, ISO 19650, EN 17412) facilitates reproducibility, interoperability, and compliance with public-sector digitalization directives. Moreover, the semi-automated implementation across FreeCAD, Revit, Dynamo, and Python demonstrates that a replicable and modular pipeline can be achieved with open or widely available software, ensuring scalability for municipal contexts. These results confirm that the proposed integration approach not only enhances the information content of road models but also transforms them into structured, AI-ready datasets suitable for predictive analysis and maintenance planning.

In accordance with recent conceptual models of DT, the multi-modal and multi-scale integration presented in this work represents the “informational backbone” of DT development. This integration ensures consistent geometry, semantic alignment across sources, and hierarchical structuring of assets—prerequisites for real-time sensing, automated model updating, and advanced decision support.

Despite these strengths, several limitations should be acknowledged.

Although the proposed system aligns with the conceptual structure of DT, it currently operates as a static information model. The integration of real-time IoT data, API-based communication with municipal platforms, and automated update routines remains beyond the present scope but represents a natural evolution of the proposed framework.

By establishing a robust, multi-source, semantically enriched data infrastructure, this study provides the foundational layer required for subsequent development of dynamic, continuously updating urban DT. The workflow was tested on a representative but limited case study (≈45 km of road network within the city of Turin). Extending the methodology to larger or morphologically different urban areas will require further validation and performance benchmarking. While the semantic model effectively integrates geometric and administrative attributes, it does not yet include behavioral or performance indicators (e.g., traffic flow, material degradation rates) that would further enhance predictive maintenance capabilities.

Although the framework presented in this study relies on periodic surveys and therefore operates as a static model, the proposed approach is intended to be compatible with future dynamic extensions. Near-real-time updates can be enabled through the integration of IoT sensors already commonly used in urban mobility and infrastructure monitoring. Examples include: traffic monitoring sensors (cameras, Bluetooth/LoRaWAN counters) that can provide flow, congestion, and event detection; environmental sensors (temperature, humidity, air quality) relevant for pavement deterioration models; structural or vibration sensors embedded in critical assets such as bridges or intersections; road-surface condition sensors, including IMU-based roughness estimation from municipal fleets or public transport vehicles.

These sensors typically stream lightweight, structured time-series data that can be associated with BIM/DT entities via stable asset identifiers. The integration of such data into the DT is technically feasible using existing IoT platforms adopted by many municipalities, and would allow the model to evolve from a static representation to a continuously updated decision-support system. Future work will therefore explore prototype integrations with selected sensors to assess data quality, update frequency, and governance implications.

Beyond these aspects, an additional set of challenges emerges when considering the scalability of the framework to an entire urban network. Automating segmentation, classification, and asset recognition is essential for managing the sheer volume and heterogeneity of city-wide datasets. In this regard, the labeled outputs produced by the current workflow provide a robust foundation for training AI models capable of addressing such complexity. Deep learning techniques, in particular, offer promising avenues for automating vertical asset detection, road marking extraction, and pavement distress identification. Their integration would significantly increase processing efficiency and reduce manual intervention, making city-scale implementations more feasible from both a time and cost perspective. AI-driven methods could also enhance the consistency of semantic labeling, support predictive maintenance strategies, and improve the identification of anomalies or missing assets across urban datasets.

At the city-scale LoD200 level, AI applications can support predictive maintenance planning and traffic safety assessment. At the high-resolution LoD400 level, AI models can enable detailed defect detection, pavement roughness analysis, and asset condition monitoring. Therefore, the proposed framework not only enhances semantic and geometric richness but also establishes a foundation for operational AI integration in municipal DT.

Another dimension that becomes increasingly relevant at municipal scale concerns data governance. Integrating datasets from different public offices, survey campaigns, and third-party providers raises issues related to data access, licensing, privacy, and interoperability. As municipalities move toward city-wide DT infrastructures, the establishment of clear governance frameworks—defining roles, update cycles, quality standards, and data-sharing agreements—will be essential to ensure the long-term usability and reliability of DT systems. The proposed workflow contributes to this direction by adopting open standards, harmonizing heterogeneous sources, and demonstrating a replicable path toward structured urban datasets that can support transparent and accountable data management.

Compared with existing DT implementations reported in the recent literature, the proposed workflow addresses a distinctive research gap. Most previous studies have focused on controlled or linear infrastructures—such as highways and bridges—where data acquisition, geometry, and semantics are relatively uniform and less challenging than in dense urban settings. For instance, Soilán et al. [18] and Davletshina et al. [21] focused on highway corridors, developing semi-automated frameworks for defining IFC alignment entities and maintaining geometric DTs. Sanfilippo et al. [33] and Tita et al. [53] explored DT applications for tunnels and bridges, emphasizing structural monitoring and lifecycle management, whereas Sofia et al. [35] demonstrated MMS-based DTs for single infrastructure assets such as the Mohammed VI bridge. Only a few works—such as Nokkaew et al. [36] and Mushtaq et al. [39]—have attempted to extend DT frameworks to urban road segments, typically limited to small-scale case studies or construction monitoring scenarios. To contextualize our methodology, we compared its performance with several existing studies of road DTs from the literature (Table 8). While direct quantitative comparison is limited by differences in reported metrics, the comparison highlights strengths and trade-offs of our approach. Compared to these, our approach delivers centimeter-level geometric accuracy, multi-level semantic enrichment (via IFC-4.3), and AI-ready labeled datasets at city scale, balancing both high fidelity and operational scale.

The reviewed applications exhibit strong contrasts across spatial scale, data resolution, accuracy, automation maturity, semantic richness, and operational value.

Coverage ranges from highly localized environments (e.g., a single bridge of 500 m or a 2 km tunnel) to very large infrastructures, such as a 500 km highway network [17] or an entire city-wide road system spanning 1434 km [38]. The Turin case (~45 km) occupies an intermediate position, representing an urban-scale MMS acquisition that balances area coverage with detail.

Significant variation is observed in data density. The highest density is reported by [35], which achieves approximately 8000 pts/m^2^ using a Leica Pegasus system at highway speed, while UAV-based acquisitions [39] produce only medium-density point clouds. Typical MMS acquisitions [17,18] report 1500–4000 pts/m^2^. The Turin dataset (1500 pts/m^2^) is comparable to standard MMS surveys for urban pavements but lower than specialized structural monitoring studies.

Accuracy varies strongly according to platform and application type. Bridge-scale MMS [35] provides the highest geometric accuracy, with RMS errors of 15–20 mm, whereas large-scale highway surveys [17,18] typically report 50 mm accuracy. City-scale mapping [38] performs slightly better, with 6–9 cm RMS. The Turin study achieves a survey accuracy of 1–3 cm, outperforming most large-scale MMS applications and reflecting the controlled conditions of an urban environment.

Automation levels range from manual or partially automated workflows [33,53] to advanced ML-driven processes [17], which integrate clustering, visual recognition, and decision-making modules. The Turin dataset adopts a semi-automated workflow but includes AI-ready semantic labels, positioning it between manual structural monitoring and fully automated highway-scale systems.

Semantic richness is another strong differentiator. The Turin case exhibits one of the highest semantic depths, using IFC 4.3 with attributes for material, type, and maintenance. Similarly, ref. [17] uses UML models enriched with pavement condition indicators (IRI, SFC). In contrast, TLS-based tunnel monitoring [33] and bridge-scale models [53] include fewer attributes, reflecting their focus on geometry rather than semantic information integration.

Operational outcomes follow the same pattern:

- Large-scale datasets [17,38] enable network-level planning and decision-support workflows.
- Bridge and tunnel studies [33,35,53] enhance structural assessment and predictive maintenance.
- The Turin dataset provides strong interoperability and downstream AI integration, supporting DT development for urban road assets.

Overall, the contrastive evaluation highlights how scale, density, accuracy, automation, and semantics combine to shape the operational value of each study. The Turin case stands out for its balance of high accuracy, semantic depth, and DT-oriented workflows, while other cases specialize in either scalability [17,38] or high-precision structural monitoring [35].

**Figure 12:**
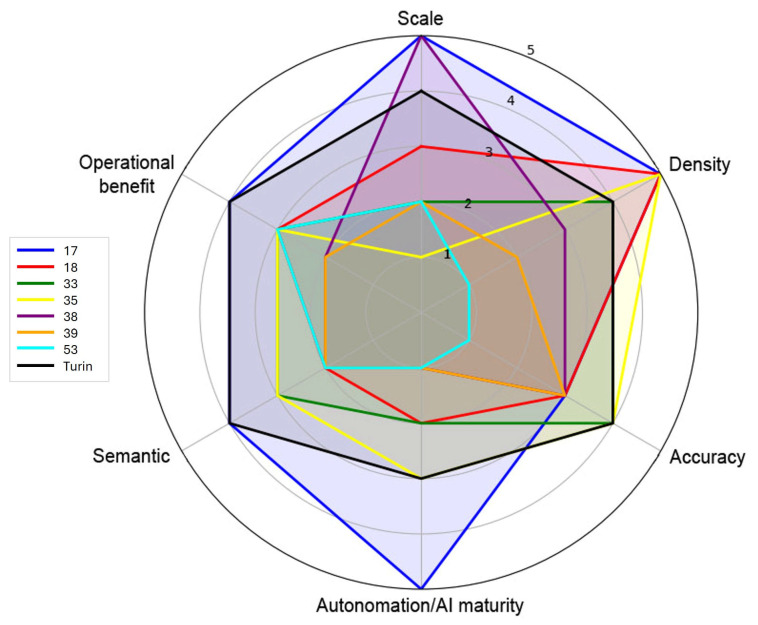
Radar plot illustrating the contrastive assessment of the selected studies. Each profile is visualized with a unique color assigned by the Matplotlib 3.9.0 default cycle. Scores for the six dimensions range from 1 (low) to 5 (high), derived from the qualitative descriptions, accuracy levels, automation approaches, and semantic richness reported in Table 8.

To synthesize the contrasts observed among the reviewed studies, a comparative radar plot was generated (Figure 12). The figure provides a normalized visualization of the six key dimensions analyzed—scale, point cloud density, accuracy, automation level, semantic richness, and operational benefit—allowing the relative strengths and limitations of each case to be directly compared. By placing all studies within a common multidimensional framework, the radar plot highlights the diverse operational profiles of DT-based workflows, ranging from large-scale, highly automated highway applications to small-scale, high-precision structural monitoring. The positioning of the Turin case illustrates its balanced configuration, with above-average accuracy, high semantic richness, and strong DT-readiness, distinguishing it from both large-scale but lower-accuracy deployments and highly precise but non-scalable asset-specific surveys. Overall, the figure visually reinforces the findings of the contrastive analysis and supports the identification of methodological gaps and complementarities across the current literature.

To address the heterogeneity of objectives, platforms, and evaluation criteria across the reviewed studies, a second level of quantitative synthesis is introduced in Table 9. Rather than directly comparing raw and inconsistently reported metrics, Table 9 aggregates the indicators presented in Table 8 into three system-level performance dimensions—efficiency, accuracy, and information completeness—using normalized scores. This strategy enables a more coherent comparison of road digitalization and DT construction workflows while avoiding unsupported algorithm-level benchmarking under non-uniform data and task conditions. In this study, *efficiency* represents the capability of a workflow to acquire, process, and manage road infrastructure data at scale, and is derived from a combination of spatial coverage, point cloud density, processing effort, and degree of automation. *Accuracy* reflects the reported geometric reliability of the acquired data or resulting models, based on survey- or model-level accuracy values provided in the original studies. *Information completeness* describes the extent to which semantic attributes are integrated into the resulting models, including asset classification, material information, condition or maintenance state, and compliance with BIM/IFC standards. Scores are assigned on a normalized 1–5 scale to enable system-level comparison across heterogeneous applications. For example, workflows covering hundreds of kilometers with high automation but limited semantic modeling score high in efficiency but lower in information completeness, whereas DT-oriented approaches prioritize semantic depth at the cost of increased processing effort.

It should be emphasized that the scores reported in Table 9 do not represent algorithmic performance metrics, but rather a normalized, system-level synthesis derived from heterogeneous indicators reported in the literature. They are intended to support comparative interpretation of workflow capabilities rather than strict benchmarking.

The quantitative synthesis presented in Table 9 provides a higher-level perspective on the comparative performance of existing road digitalization and DT workflows. By grouping heterogeneous indicators into efficiency, accuracy, and information completeness dimensions, the analysis highlights systematic trends that are not evident from isolated metrics alone. In particular, large-scale MMS-based approaches generally achieve high efficiency scores due to extensive spatial coverage and high acquisition density, while their semantic richness remains limited when compared to DT-oriented workflows. Conversely, approaches explicitly designed for digital twin construction, such as the Turin case study, achieve higher information completeness scores through structured semantic modeling, IFC compliance, and the explicit representation of maintenance and condition attributes, albeit at the cost of increased processing complexity.

Importantly, this analysis confirms that current road DT research lacks a unified quantitative evaluation framework. Reported metrics are often incomplete, non-standardized, and strongly dependent on application context, acquisition platform, and study objectives. As a result, the comparison presented here should be interpreted as a system-level assessment of workflow capabilities rather than a strict performance benchmark of individual algorithms. A fully controlled comparison of method performance under identical data and task conditions is currently infeasible, as no shared benchmark datasets or standardized evaluation protocols exist for urban road DT construction.

These findings highlight the need for standardized datasets and evaluation protocols to enable reproducible quantitative benchmarking in future road DT research.

The present study develops a fully integrated BIM–GIS methodology specifically designed for complex urban environments, applying it to a large and heterogeneous portion of Turin’s road network. This city-scale perspective introduces both interoperability advantages—through the use of open standards such as IFC 4.3—and new challenges related to data harmonization across varying resolutions, coordinate systems, and semantic structures. By demonstrating a scalable, multi-source, and multi-level integration pipeline, the proposed workflow advances current DT research toward operational, municipal-scale DTs capable of supporting decision-making, monitoring, and predictive maintenance in real urban settings.

In practical terms, this integration offers municipalities a pathway to transition from fragmented 2D mapping systems to comprehensive 3D information environments. The methodology can be extended to other urban infrastructures—such as drainage networks, or underground services—using the same multi-scale, open-standard framework. Future work will focus on automating point cloud feature extraction through AI-based or deep learning techniques, integrating IoT-based real-time data streams for dynamic updates, and exploring governance frameworks to standardize DT adoption at municipal and regional levels.

The practical value of the proposed DT-ready model lies in its capacity to support urban asset monitoring and maintenance tasks. By harmonizing MMS-derived geometry with municipal geospatial records, the model enables consistent asset inventories, automated completeness checks, spatial verification of municipal registers, and the identification of missing or misaligned elements. The reconstructed pavement surfaces serve as a geometric foundation for pavement condition analyses, including AI-based defect detection and future integration with road roughness sensors. Moreover, by assigning unique identifiers and semantic attributes to each asset, the model facilitates interoperability with municipal maintenance systems, scheduling tools, and urban management dashboards.

In this perspective, the proposed workflow also lays the foundation for the creation of a standardized urban dataset of the Turin road network, comparable to initiatives such as the CAMHighways dataset developed by Digital Future Roads in Cambridge [37]. Establishing such a publicly accessible, semantically enriched dataset would represent both an academic and practical advancement, providing a benchmark for AI-based research on road condition assessment, infrastructure monitoring, and DT interoperability. Beyond its local relevance, this dataset could serve as a reference model for future city-scale DT implementations, fostering open, data-driven management of urban road systems.

Ultimately, these developments will enable more resilient, data-driven, and sustainable management of urban transport infrastructure.

## 5. Conclusions

This study presented a BIM-based DT framework for urban road networks that integrates high-resolution MMS-derived LiDAR point clouds with municipal GIS layers and authoritative geodatabases. The proposed multi-source and multi-scale workflow enables accurate geometric reconstruction at LoD200 and LoD400 levels and semantic enrichment through the IFC 4.3 schema, ensuring full interoperability between BIM and GIS environments. By combining geometric precision with semantic depth, the resulting DT provides a comprehensive, machine-readable representation of the urban road infrastructure.

The framework demonstrates that existing municipal and survey data can be effectively merged to produce a standardized, AI-ready model that supports both city-level analyses and detailed asset management. The achieved geometric accuracy (RMSE ± 3 cm), adherence to open standards and reproducibility using accessible software platforms confirm the technical robustness and practical scalability of the approach.

The outputs generated by the workflow—including structured asset inventories, semantically enriched BIM objects, and reconstructed pavement geometries—provide actionable information for monitoring and maintenance operations. These results highlight the practical relevance of the DT-ready environment. The proposed framework delivers multi-modal, multi-scale, and multi-level integration that forms the foundational layer of a future DT, while fully sensor-integrated update mechanisms remain a subject for future development.

Despite these promising results, several limitations remain. First, full automation of point cloud segmentation and asset classification is still challenging, especially in dense urban environments affected by occlusions, variable point density, and object ambiguity. Second, the current DT is static, relying on periodic surveys rather than continuous IoT ingestion, which limits its real-time decision-support capabilities. Third, the scalability of the framework to an entire metropolitan area is conditioned by computational demands, data coordination efforts, and institutional capacity.

A further and particularly significant limitation concerns data governance, policy constraints, and interoperability between institutional datasets. Integrating municipal geodatabases, survey data, and third-party sources requires navigating heterogeneous data standards, incomplete metadata, and varying update cycles. Moreover, issues related to privacy, licensing, access restrictions, and long-term data stewardship may hinder the consolidation of a unified city-level DT. This study also highlights a broader limitation in current road digitalization and DT research: the absence of standardized datasets, evaluation tasks, and quantitative benchmarks. Future research will address this gap by developing shared reference datasets, defining task-specific performance metrics, and enabling controlled comparisons of extraction, modeling, and semantic enrichment methods under identical conditions—an essential step toward reproducible, algorithm-level evaluation.

These challenges emphasize the need for shared protocols for data acquisition, management, and lifecycle control, as well as coordinated policies between municipal authorities and research institutions.

At the same time, the proposed workflow can help address some of these governance challenges. By structuring all outputs through open standards (IFC 4.3, GeoPackage, EPSG-compliant CRS) and producing fully traceable, machine-interpretable datasets, the method supports transparent data exchange and reduces dependency on proprietary formats. The explicit linking of survey-derived features with authoritative geodatabases also strengthens institutional data consistency and can facilitate future policy development around open-data initiatives, quality assurance, and long-term digital asset management.

Overall, while the current framework does not yet constitute a fully dynamic DT, it provides a solid, standardized, and scalable foundation upon which real-time sensing, automated processing, and advanced decision-support systems can be built. The methodology offers a replicable pathway for municipalities aiming to implement operational DTs and lays the groundwork for a unified, semantically enriched dataset of the Turin road network. Such a resource will foster further research on AI-based infrastructure assessment and support data-driven, sustainable management of the urban road system.

## Figures and Tables

**Table 1:** Representative studies on DT applications in road infrastructure.

Reference	Data Source–Sensors	Scale–Environment	Application Focus	Main Contribution
[31]	LiDAR, UAV, SNOET	Suburban Segment	Management process	Highlight the combination of environmental data and physical data to assist maintenance.
[32]	ALS, MLS	Highway Segment	Planning and management	Highlight the path from reality capturing towards the establishment of the digital representation as-is of existing roads.
[33]	TLS	Highway Tunnel	Operation and maintenance	Development of a DT to explore how to use it for management and maintenance activities.
[34]	Geodatabase	Ring Road	Operation and maintenance	Highlight the challenges of interoperability between BIM and GIS.
[35]	MMS	Highway Bridge	Monitoring and maintenance	Development of a DT platform for monitoring and maintaining cable bridges.
[36]	IoT, GPS, UAV	Urban Intersection	Traffic analysis	Provides a data fusion idea for digital twin systems in ITS and cross integration analysis.
[37]	MMS, GPR	Highway	Inspection and maintenance	Building of a highway DT enhancing inspection and maintenance processes.
[38]	MMS	Urban segment	Real time analysis	Highlight effectiveness of MMS-derived data in producing highly accurate DT for smart city applications.
[39]	UAV	Urban segment	Monitoring of road segment construction	Integration of BIM and photogrammetry in the specific context of road infrastructure construction.
[40]	LiDAR, GPS Camera, IoT	Local Roads and Bridge	Monitoring and maintenance	Development of an integrated DT framework combining BIM and IoT sensor data to enable real-time monitoring, analysis, and maintenance.
[41]	IMU, Camera	Urban and suburban roads	Maintenance and management	Development of DT for real-time pavement condition evaluation.

**Table 2:** Road segments characteristics derived from municipal geodatabase.

Type	Number of Segments	Total Surface (m^2^)	Average Width (m)	Lanes	Type of Surface
Arterial	12	502,494	13–15	2–3	Asphalt
Secondary	24	584,657	10–12	2	Asphalt
Local	187	774,994	5–7	1	Asphalt or Stone
Intersection	123	497,686	-	-	Asphalt or Stone

**Table 3:** Overview of BIM Levels of Development applied in the study.

LoD	Scale	Primary Data Sources	Purpose/Usage	Information and Detail Level
200	City-scale/entire study area	GIS layers (CTC), LiDAR-derived pavement and vertical asset info	General overview, multi-source data fusion, GIS integration	Simplified geometry of pavements, sidewalks, vertical assets; enriched semantic attributes.
400	Local-scale/selected road segments	MMS LiDAR point clouds, GIS layers, authoritative geodatabase	High-resolution monitoring, analysis, and management of critical infrastructure	Detailed pavement geometry, high-fidelity reconstruction of vertical assets, enriched semantic attributes.

**Table 4:** Processing time for the proposed workflow.

Workflow Stage	Approximate Duration
MMS data acquisition	2 day
Raw point cloud processing	4–5 days
Classification	2 day
Feature clustering	1 day
Bim parametric modelling LoD200	1 day
Bim parametric modelling LoD400	1 day

**Table 5:** BIM element and IFC schema hierarchy.

Asset	BIM Element	IFC
Horizontal	Road segment	IfcRoad
	Sidewalk	IfcPavement
	Road marker	IfcSign
Vertical	Vertical sign	IfcSign
	Traffic light	IfcSignal
	Streetlight	IfcLightFixture

**Table 6:** Comparison of geometric, semantic, and operational characteristics of the individual data sources and the integrated BIM-DT.

Source	Geometric Detail	Spatial Accuracy	Semantic Attributes	Temporal Update	AI-Readiness
LiDAR point cloud	Very High (1500 pts/m^2^)	±1 cm	None	Survey-based	Unstructured
Municipal Geodatabase	Low (2D polygon)	±20 cm	High (class, material, maintenance)	Periodic	Unstructured
Integrated BIM-DT	High (LoD 200/400)	±3 cm	Very High (geometric + semantic)	Updatable	Structured

**Table 7:** Potential AI applications.

Asset Type	Number of Instances	Key Geometric Parameters	Semantic Attributes	AI—Application
Streetlight	1178	Height, diameter, location	Material, maintenance zone, ownership	Automated recognition, condition assessment, predictive maintenance
Traffic Light	236	Height, diameter, location	Material, functional class, maintenance status	Automated recognition, condition assessment, predictive maintenance
Vertical Sign	932	Height, diameter, location	Sign type, material, installation date	Traffic sign recognition, visibility analysis, maintenance scheduling
Pavement Surface	45 km	3D surface geometry	Material, functional class, maintenance zone	Pavement roughness analysis, defect detection, deterioration prediction

**Table 8:** Comparison with existing representative DT studies. The compared studies deploy different sensors, targets (highway vs. urban roads vs. tunnels), and reported metrics. The table focuses on comparable qualitative aspects. NR, Not Reported.

Ref.	Scale/ Coverage	Point Cloud/Data Density	Accuracy (Reported or Typical)	Autonomation/AI Level	Semantic/ Attribute Richness	Operational Benefit
Turin	~45 km urban network	High-Density MMS, 1500 pts/m^2^ on road pavements	Survey accuracy ~1–3 cm; 3D Model accuracy ± 3 cm	Semi-automated; labeled for AI	IFC-4.3 with material, type, maintenance attributes	AI-ready data, semantic interoperability, decision support potential
[17]	Highway-scale application (~500 km)	High-density MMS, KAARTA Stencil 2 typical point density at 60 km/h ~4000 pts/m^2^	Typical MMS Survey accuracy ~5 cm; 3D Model accuracy NR	High automation; ML-based clustering + Computer Vision, DT-driven DSS workflows	Semantic UML-based model attributes include IRI, SFC, inspections, condition classes	Large-scale planning; Decision support system
[18]	~20 km of highway road	High-Density MMS, 155 million points covers 17.5 km	Typical MMS Survey accuracy ~5 cm	Semi-automated; manual intervention needed	Road alignment & markings, IFC-based entities	Useful for asset management; limited scalability for entire networks
[33]	Tunnel-scale (~2 km)	High-Density TLS	Survey accuracy ± 3 cm; 3D Model accuracy NR	Manual and partial automation	Tunnel elements and maintenance workflows	Improves operation and maintenance scheduling for tunnels
[35]	Single bridge (~500 m)	High-Density MMS, Leica Pegasus two typical point density at 60 km/h ~8000 pts/m^2^	Survey horizontal accuracy 0.02 m RMS, vertical accuracy 0.015 m RMS; 3D Model accuracy NR	ML-assisted monitoring; partially automated	Bridge and road asset monitoring; condition assessment	Facilitates predictive maintenance and asset monitoring
[38]	City-scale road network (1434 km)	High-Density MMS	Survey horizontal accuracy 0.06 m RMS, vertical accuracy 0.09 m RMS, 3D Model accuracy NR	Conceptual DT; low automation	Broad infrastructure categories (roads, signals)	Smart-city modeling; web visualization
[39]	Small urban network (~1–2 km)	Medium-Density UAV	RTK centimeter accuracy, 3D Model accuracy NR	Photogrammetry-assisted semi-automated	IFC-based road assets; some attribute enrichment	Case-study DT for urban road management; moderate operational value
[53]	Bridge-scale (~1 km)	NR	NR	BIM-DT integration; mostly manual	Bridge structural & maintenance data	Supports bridge lifecycle management; decision-support tool

**Table 9:** Normalized system-level quantitative comparison of road digitalization and digital twin workflows across efficiency, accuracy, and information completeness dimensions. Scores (1–5) are derived from reported values in the original studies.

Reference	Efficiency Score	Accuracy Score	Information Completeness Score
Turin	4	5	5
[17]	5	3	5
[18]	4	3	3
[33]	2	4	2
[35]	3	5	3
[38]	5	2	2
[39]	3	3	3
[53]	1	1	2

## Data Availability

The raw data supporting the conclusions of this article will be made available by the authors on request.

## Abbreviations

The following abbreviations are used in this manuscript:

DT	Digital Twin
BIM	Building Information Modeling
IFC	Industry Foundation Class
GIS	Geographic Information System
AI	Artificial Intelligence
MMS	Mobile Mapping System
LiDAR	Light Detection and Ranging
UAV	Unmanned Aerial Vehicle
IoT	Internet of Things
GPS	Global Positioning System
GPR	Ground Penetrating Radar
IMU	Inertial Measurement Unit
ALS	Airborne Laser Scanning
TLS	Terrestrial Laser Scanning
SNOET	SNiffing Omgewing/Environmental Tester
LoD	Level of Development
RMSE	Root Mean Square Error
CRS	Coordinate Reference System
EPSG	European Petroleum Survey Group
CTC	Carta Tecnica Comunale
DBSCAN	Density-Based Spatial Clustering of Applications with Noise
